# MeCP2 suppresses LIN28A expression *via* binding to its methylated-CpG islands in pancreatic cancer cells

**DOI:** 10.18632/oncotarget.7507

**Published:** 2016-02-19

**Authors:** Min Xu, Shihui Bian, Jie Li, Junbo He, Hui Chen, Lu Ge, Zhijun Jiao, Youli Zhang, Wanxin Peng, Fengyi Du, Yinyuan Mo, Aihua Gong

**Affiliations:** ^1^ Department of Gastroenterology, Affiliated Hospital of Jiangsu University, Jiangsu University, Zhenjiang, China; ^2^ Department of Pharmacology Toxicology and Cancer Institute, University of Mississippi Medical Center, Jackson, MS, USA; ^3^ Department of Cell Biology, School of Medicine, Jiangsu University, Zhenjiang, China

**Keywords:** LIN28A, CpG islands, MeCP2, pancreatic cancer

## Abstract

LIN28A aberrant expression contributes to the development of human malignancies. However, the LIN28A expression profile remains to be clarified. Herein, we report that LIN28A expression is directly associated with the methylation status of its two CpG island sites in pancreatic cancer cells. First, Bisulfite sequencing reveals that PANC1 cells possess the higher methylation rate at LIN28A CpG islands compared with SW1990 and PaTu8988 cells. Subsequently, LIN28A expression is increased at both mRNA and protein levels in pancreatic cancer cells treated with 5-Aza-2′-deoxycytidine (5-Aza-CdR), a DNA methyltransferase inhibitor. Further Chromatin immunoprecipitation (ChIP) assays indicate that methyl-CpG-binding protein 2 (MeCP2) binds preferentially to the two hypermethylated CpG islands sites at LIN28A promoter compare to MBD3. Expectedly, MeCP2 knockdown transcriptionally activates LIN28A expression in above cells, rather than MBD3 knockdown. Moreover, LIN28A overexpression remarkably improves OCT4, NANOG and SOX2 expression, and the ability of sphere and colony formation, and enhances the capacities of invasion in PaTu8988 and SW1990 cells, whereas LIN28A knockdown significantly inhibits the above malignant behaviors in PANC1 cells. These findings suggest that LIN28A is epigenetically regulated via MeCP2 binding to methylated-CpG islands, and may play a crucial role in pancreatic cancer progression.

## INTRODUCTION

LIN28A, a highly conserved RNA-binding protein, plays an important role in inducing pluripotent stem cells, regulating development, cell growth, and metabolism [1-4]. Emerging evidence has shown that LIN28A is over-expressed in a variety of human tumors including oesophagus cancer [5], oral squamous cancer [6], colon cancer [7], and epithelial ovarian cancer [8]. Furthermore, LIN28A-positive tumors are poorly differentiated and more aggressive [5, 9, 10]. However, the profile of LIN28A expression remains to be clarified.

DNA methylation, an epigenetic regulation way, controls gene expression by recruiting proteins involved in transcriptional repression [11]. Previous studies have identified that Methyl-CpG binding proteins (MeCPs) recruit chromatin remodelers, histone deacetylases, and methylases to methylated DNA, leading to transcriptional repression [12]. For example, transcriptional repressor Methyl-CpG-binding Protein 2 (MeCP2) and Methyl-CpG-binding domain 2 (MBD2) play a crucial role in CpG island methylation both *in vivo* and *in vitro* [13-17]. Whether the LIN28A expression underlies epigenetic regulation mechanism remains to be clarified in pancreatic cancer cells.

In this study, we found that LIN28A expression had significant difference in pancreatic cancer cells, and was associated with the methylation status of two CpG islands sites. MeCP2 bound preferentially to the hypermethylated CpG islands to suppress LIN28A expression. We also found that LIN28A was critical for the stemness maintenance and invasion of pancreatic cancer cells. These findings for the first time prove that LIN28A expression is associated with methylation status of CpG islands, and may play a crucial role in pancreatic cancer progression.

## RESULTS

### LIN28A Expression in different types of pancreatic cancer cell lines

**Figure 1:**
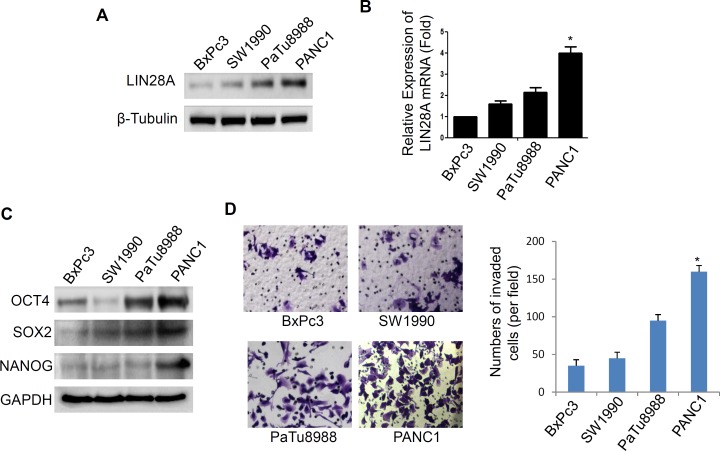
LIN28A expression in pancreatic cancer cells Relative expression levels of LIN28A protein **A.** and mRNA **B.** were assessed in BxPC3, SW1990, PaTu8988, and PANC1 cells. The stem cell makers SOX2, OCT4 and NANOG were determined by western blotting **C.** and the abilities of invasiveness were examined using transwell assay **D.** Scare bar = 50μm.

It has been reported that LIN28A expression are reactivated in human cancers [10, 18, 19]. However, the LIN28A expression profile in pancreatic cancer cells is still unknown. We analyzed the LIN28A expression in BxPC3, PANC1, SW1990 and PaTu8988 cells using real-time PCR and western blot. The results showed that LIN28A expression, at both mRNA and protein levels, was higher in PANC1 cells than that in three other cells (Figure 1A, 1B). As LIN28A is associated with the differentiation of cancer cells, we evaluated the markers of stem cells OCT4, SOX2 and NANOG, and found that their expression in PANC1 cells was higher than that of the other cells (Figure 1C, S1B), indicating that PANC1 cells possess more poor differentiation state, which is consistent with previous studies in other tumor types. Moreover, we also found that PANC1 cells were more invasive among the above cells (Figure 1D).

### Methylation status of the LIN28A CpG islands in pancreatic cancer cells

**Figure 2:**
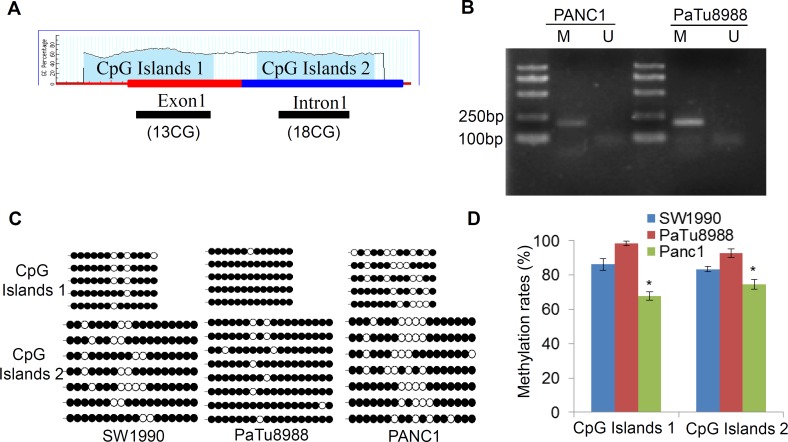
Aberrant methylation at LIN28A CpG islands in pancreatic cancer cells **A.** Schematic map of LIN28A CpG islands, Two CpG islands sequences locates at human *LIN28A* from −79 to +98 and from +139 to +406 based on the MethyPrimer Software. **B.** Bisulfite-treated genomic DNA from PaTu8988 and PANC1 cells was used for PCR amplification by specific primers. M: products (175bp) generated by primers specific for methylated DNA; U: products (173bp) generated by primers specific for unmethylated DNA. **C.** The methylation status of the individual CpG dinucleotides at human *LIN28A* CpG islands was analyzed by bisulfite sequencing in SW1990, PaTu8988, and PANC1cells. The results are shown by methylated (black) or unmethylated (white) circles. The methylation rates of both *LIN28A* CpG islands in SW1990, PaTu8988, and PANC1 cells were shown in **D.** Data are presented as mean±SD.*, *P* < 0.05.

Although LIN28A plays important roles in many kinds of tumor cells, the mechanism underlying LIN28A different expression pattern is unclear. Since methylation status of CpG within proximal promoters is often associated with transcriptional silencing, we first analyzed the predictable CpG islands of *LIN28A* promoter using the MethPrimer software. The criteria are: Island size > 100, GC Percent >50.0, Obs/Exp (Observed/Expected number of CpG patterns) ratio > 0.6. The first CpG islands were identified in the first exon from −79 bp to +98 bp, and the second CpG islands were in the first intron from +139 bp to +406 bp (Figure 2A). Therefore, we examined the methylation status of both sites in pancreatic cancer cells using bisulfite sequencing. The results indicated that both sites had different methylation rates in SW1990, PaTu8988, and PANC1 cells, with 86.15%±3.5%, 98.46%±1.5%, and 67.69%±2.5%, respectively at the first site; as well as 83.33%±1.5%, 92.85%±2.5%, and 74.60%±3% at the second site (Figure 2B-2D). Obviously, the methylation levels of both sites in PANC1 cells were lower than the other two cells, supporting the hypothesis of LIN28A epigenetic silencing via CpG islands hypermethylation.

### Re-activation of LIN28A expression by 5-Aza-CdR

**Figure 3:**
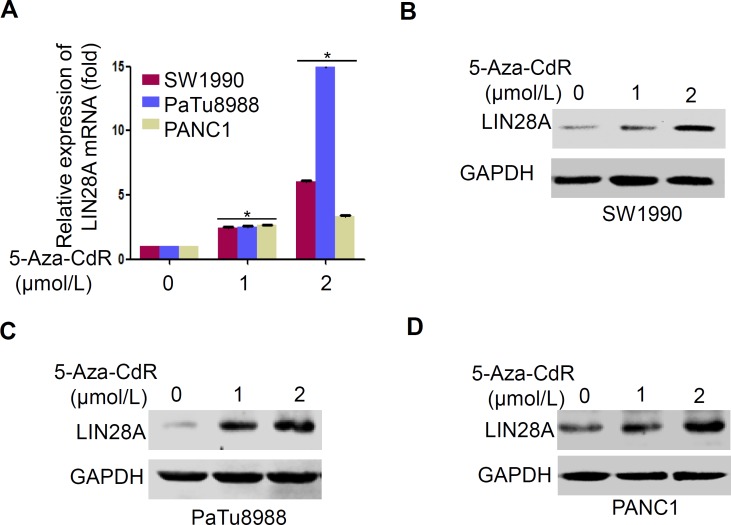
5-Aza-CdR re-activates LIN28A expression in pancreatic cancer cells **A.** Pancreatic cancer cells were treated with 5-Aza-CdR in 0, 1, 2 μmol/L for 3 days with the medium changed every day. LIN28A mRNA or protein expression was determined using real-time PCR and western blot, respectively in SW1990 **B.**, PaTu8988 **C.**, and PANC1 **D.** cells. GAPDH was used as a control. Data are presented as mean±SD.*, *P* < 0.05.

To further evaluate the role of CpG islands methylation in LIN28A expression, we subsequently treated pancreatic cancer cells with the methyltransferase inhibitor 5-Aza-2′-deoxycytidine (5-Aza-CdR). The results indicated that 5-Aza-CdR could, to different extent, induce LIN28A expression at both protein and mRNA levels in a dose-dependent manner (Figure 3A-3D). As expected, in PaTu8988 cells with higher methylation levels of CpG islands, LIN28A mRNA expression was increased over 12-fold, while only about 6-fold or 3-fold in SW1990 or in PANC1 cells, respectively (Figure 3A). Such different inductions by 5-Aza-CdR were consistent with their methylation statuses in *LIN28A* CpG islands sites. It is indirectly suggested that the higher CpG island methylation level may play a crucial role in suppressing LIN28A expression.

### MeCP2 reads the methyl-CpG islands to suppress LIN28A expression

**Figure 4:**
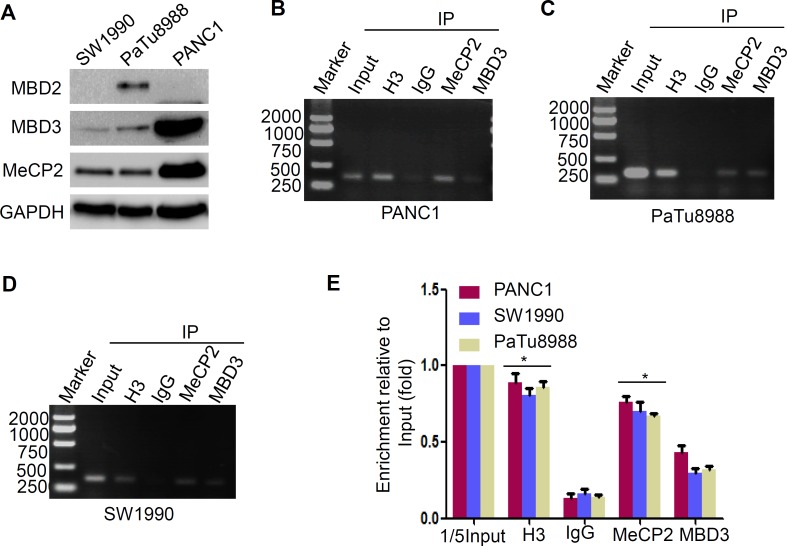
MeCP2 binds to methylated-CpG islands to suppress LIN28A expression **A.** Western blotting was used to analyze the endogenous MeCP2, MBD2 and MBD3 protein expressions in indicated pancreatic cancer cells. **B.** Cross-linked chromatins from indicated pancreatic cancer cells were incubated with antiserum against H3, IgG, MBD3, and MeCP2. DNA extracted from each immunopecipitate was analyzed by standard PCR **B.**-**D.** or Real-time PCR **E.** with primers specific for *LIN28A* CpG islands 2. Chromatin taken before immunopecipitatipn was used as ‘Input’ controls. The ‘Input’ diluted 5 times was used as a control. Each bar represents the mean±SD of three independent assays.

Methyl-CpG binding domain (MBD) proteins recruit repressing proteins to the methylated DNA, leading to transcriptional suppression. Previous studies identify that MeCP2 and MBD2, specifically binding to a single methyl-CpG pair, are the founding members of the MBD protein family [21], while MBD3 is found at unmethylated CpG islands and active promoters [22]. To determine whether MeCP2 or MBD2 contributes to promoter CpG island methylation-mediated suppression of LIN28A expression, we firstly detected the expression levels of MeCP2, MBD2 and MBD3 in the above three cells, and found that expression of MeCP2 and MBD3 was higher in PANC1 cells compared with the other two cells, while MBD2 was weakly expressed only in PaTu8988 cells (Figure 4A). It is implied that MeCP2 might be a methyl-CpG readers involved in suppression of LIN28A expression. Therefore, we performed ChIP assays to examine the readers in PaTu8988 and PANC1 cells using MeCP2 antibody, and MBD3 antibody as a negative control. Expectedly, MeCP2 could bind to hypermethylated CpG islands in *LIN28A* promoter, but weakly MBD3 (Figure 4B-4D) in three above cells. The analysis of relative quantitation was shown in (Figure 4E). Our data further confirmed the previous MeCPs reading pattern, and suggested that MeCP2, as methyl-CpG readers to *LIN28A* promoter, might be strongly involved in LIN28A epigenetic silencing in pancreatic cancer cells.

### MeCP2 regulates LIN28A transcriptional suppression

**Figure 5:**
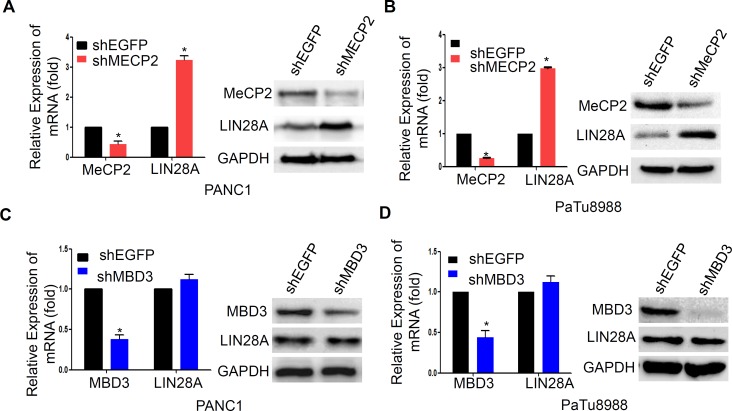
MeCP2 knockdown re-activates LIN28A expression LIN28A mRNA and protein levels were detected by Real-time PCR or western blotting in PANC1 **A.** and PaTu8988 **B.** cells transfected with sh-MeCP2 or sh-EGFP plasmids, as well as MBD3 knockdown in PANC1 **C.** and PaTu8988 cells **D.** Data are presented as mean±SD. *, *P* < 0.05.

To further confirm the role of MeCP2, as a methyl-CpG reader, in epigenetic regulation of LIN28A expression, we transfected PaTu8988 and PANC1 cells with sh-MeCP2 or sh-MBD3 to examine LIN28A expression at both protein and mRNA levels, and sh-GFP as a control. Actually, MeCP2 knockdown significantly resulted in up-regulation of LIN28A expression in PANC1 and PaTu8988 cells compare to control group (Figure 5A, 5B), while MBD3 knockdown had no effect on LIN28A expression (Figure 5C, 5D). This further confirmed that MeCP2 contributed to epigenetic regulation of LIN28A expression in pancreatic cancer cells.

### LIN28A improves the ability of stemness maintenance in pancreatic cancer cells

**Figure 6:**
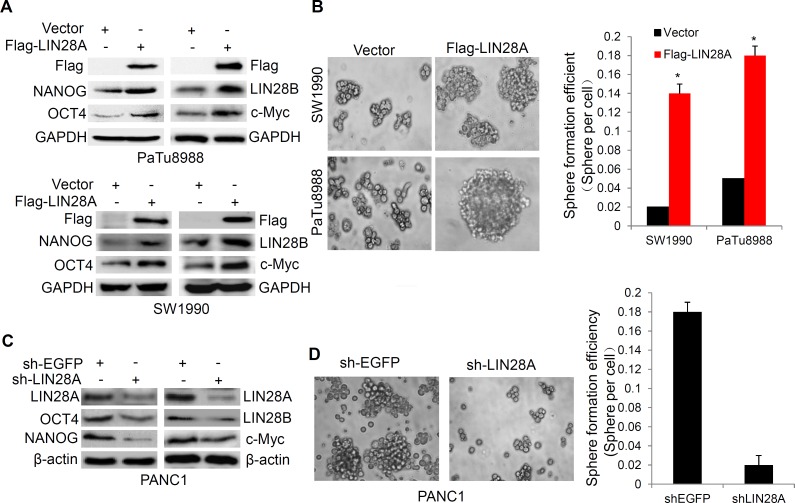
LIN28A promotes the expression of stem cell makers and sphere formation in pancreatic cancer cells **A.** The stem cell makers OCT4, NANOG, LIN28B and c-myc expression were examined using western blotting in PaTu8988 and SW1990 cells transfected with Flag-LIN28A or Vector plasmids, and sphere formation efficiencies were shown in **B. C.** PANC1 cells were transfected with sh-LIN28A or sh-EGFP plasmids, and the stem cell makers OCT4, NANOG, LIN28B and c-myc expression were examined using western blotting, and sphere formation efficiencies were shown in **D.** Data are presented as mean±SD. *, *P* < 0.05.

To clarify whether LIN28A expression is associated with stemness maintenance of pancreatic cancer cells, LIN28A or Vector were overexpressed in PaTu8988 and SW1990 cells, we found that LIN28A promoted OCT4, NANOG, LIN28B and c-myc expression (Figure 6A), as well as the sphere formation and colony-forming ability (Figure 6B and Figure S2). In contrast, Knocking down LIN28A resulted in the opposite effects in PANC1 cells (Figure 6C and Figure S2), suggesting that LIN28A was significantly involved in the stemness maintenance of pancreatic cancer cells.

### LIN28A enhances the invasion ability of pancreatic cancer cells

**Figure 7:**
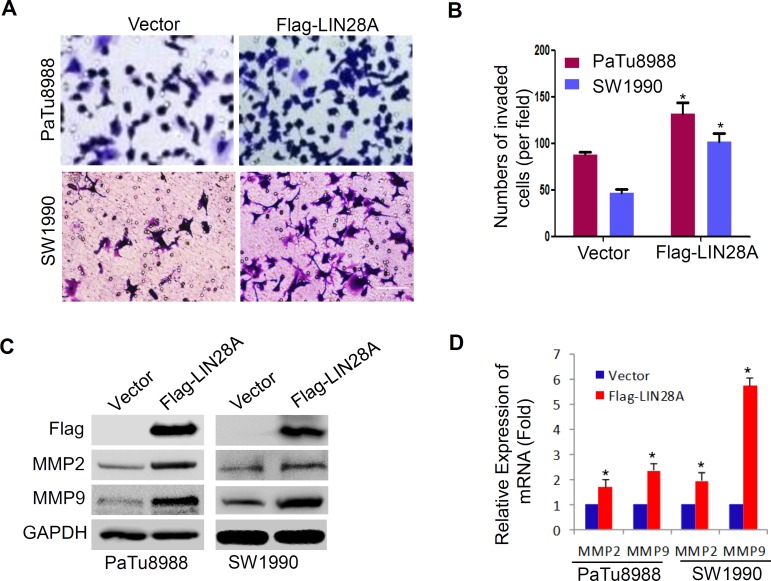
LIN28A promotes the invasiveness in pancreatic cancer cells **A.** The abilities of invasiveness were examined using transwell assay in PaTu8988 and SW1990 cells transfected with Flag-LIN28A or Vector plasmids. Invaded cells were counted and analyzed in **B.** MMP2 and MMP9 were determined using western blotting **C.** and real-time PCR **D.** in above cells. Data shown are mean±SD of three triplicate measures. *, *P* < 0.05.

To assess the role of LIN28A in human pancreatic cancer cells, we further examined the abilities of invasion by transwell assays. PaTu8988 and SW1990 cells were transfected with Flag-LIN28A or Vector plasmids, and PANC1 cells with sh-LIN28A or sh-GFP plasmids for 72h. We found that LIN28A overexpression significantly promoted the invasion ability of PaTu8988 and SW1990 cells (Figure 7A, 7B), whereas LIN28A knockdown inhibited the invasion ability of PANC1 cells (Figure S3A, B). Also, LIN28A overexpression resulted in upregulation of MMP2 and MMP9 (Figure 7C, 7D), while LIN28A knockdown downregulated the expression of MMP2 and MMP9 in PANC1 cells (Figure S3 C, D), indicating that LIN28A might be critical for invasion of pancreatic cancer cells.

## DISCUSSION

Herein, we identify that LIN28A is required for the differentiation of pancreatic cancer cells, and promotes their growth and invasion. Mechanically, we for the first time identify that LIN28A expression is associated with CpG islands methylation status of its promoter, and epigenetically regulated by MeCP2, a methyl-CpG reader, in pancreatic cancer cells. These findings suggest that LIN28A might be a candidate of pancreatic cancer biomarkers.

The pluripotency factors, such as NANOG, SOX2, OCT4, and LIN28A, suggest a close relationship between tumor formation and the process of reprogramming mature cells to a more primitive type. LIN28A and LIN28B are specifically activated in the subset of tumors that are poorly differentiated and carry the worst prognosis [23]. In this study, we find that LIN28A increases the expression of stem cell markers SOX2, OCT4, LIN28B, c-Myc and NANOG in pancreatic cancer cells, and promotes their malignant behaviors including growth and invasion *in vitro*, further supporting the oncogenic potential of LIN28A. Previous studies have revealed that c-Myc- LIN28A/LIN28B forms a positive feedback loop [4, 24, 25]; it is noted whether the other pluripotency factors such as LIN28A-self are involved in such positive feedback loop.

Genetic alterations and epigenetic alterations are the main features in the pathogenesis of pancreatic cancer. In epigenetics, it has been proposed to define the differential methylated genes between pancreatic cancer cells and normal pancreatic tissues [26]. In this study, we at the first time find that LIN28A expression is associated with methylation status of the *LIN28A* promoter-associated CpG islands. It has reported that direct binding of MeCPs to the methylated DNA plays a crucial role in gene suppressing [27, 28]. Previous studies identify that MeCP2 and MBD2, specifically binding to a single methyl-CpG pair, are the founding members of the MBD protein family [21], while MBD3 is found at unmethylated CpG islands and active promoters [22]. Our ChIP assays reveal that MeCP2 effectively binds to hypermethylated *LIN28A* CpG islands, rather than MBD3, especially in PaTu8988 cells. Furthermore, the *LIN28A* transcriptional activity is restored after MeCP2 knockdown in both PaTu8988 and PANC1 cells, but not silencing of MBD3. These findings confirm that MeCP2 suppresses LIN28A transcriptional activity via reading methylated CpG islands.

In summary, our data reveals that high expression of LIN28A may be related to the malignant behaviors of pancreatic cancers, and MeCP2 directly binds to Methylated-CpG islands for LIN28A transcriptional suppression in pancreatic cancer cells. These findings suggest that LIN28A might be an important therapeutic target candidate to pancreatic cancers.

## MATERIALS AND METHODS

### Cell culture

The pancreatic cancer cell lines BxPC3, SW1990, PaTu8988 and PANC1 were kindly provided by Second Military Medical University in shanghai, and cultured with DMEM (Hyclone, China) supplemented with 10% fetal bovine serum (Gibco, USA), 100mg/L penicillin, and 100mg/L streptomycin, in humidified 5% CO_2_ incubator at 37°C.

### Real-time PCR

Total RNA was isolated using RNAiso Plus (Takara). Reverse transcription was performed using RevertAid First Strand cDNA Synthesis Kit (Thermo) according to the manufacturer's recommendations. The SYBR green-based Real-time PCR was then performed in triplicate using CFX-96 Sequence Detection System (Bio-Rad) and gene expression was normalized by GAPDH. Primers were list in Table 1. The relative fold change in RNA expression was calculated using the 2^−^ΔΔCt method.

**Table 1:** DNA and RNA nucleotide sequences

LIN28-F	CGCTCGACCCCCCAGTGGATG
LIN28-R	TGGGGTGGCAGCTTGCATTCC
GAPDH-F	TGGGGAAGGTGAAGGTCGG
GAPDH-R	CTGGAAGATGGTGATGGGA
MeCP2-F	CAGCGTCTGCAAAGAGGAGA
MeCP2-R	GCTCCTCTCTGTTTGGCCTT
BSP-F1	GGGGGAAGATGTAGTAGTTTTTTTT
BSP-R1	CCTTTAAACAACCTAAAACTCAATTC
BSP-F2	TTAGGTTGTTTAAAGGATTTTAAGA
BSP-R2	AAACAACTACCAAACAACAAAAAAAA
ChIP-PCR-F	GAACCAACCCTTTGC
ChIP-PCR-R	AAAGTGTCCCCGCTAAGTCC
LIN28-all-F	CGGGGTACCGGCTCCGTGTCCAACCAG
LIN28-all-R	CGCGGATCCATTCTGTGCCTCCGGGAG
ShMeCP2-F	CCGGCGTCTGCAAAGAGGAGAAGATCTCGAGATCTTCTCCTCTTTGCAGACGTTTTTG
ShMeCP2-R	AATTCAAAAACGTCTGCAAAGAGGAGAAGATCTCGAGATCTTCTCCTCTTTGCAGACG
SheGFP-F	CCGGTACAACAGCCACAACGTCTATCTCGAGATAGACGTTGTGGCTGTTGTATTTTTG
SheGFP-R	AATTCAAAAATACAACAGCCACAACGTCTATCTCGAGATAGACGTTGTGGCTGTTGTA

### Western blotting

The cultured cells were rinsed with cold PBS before treated with RIPA lysis buffer at 4°C for 10 min. Then the mixture was centrifuged under 4°C at 12000r/min for 15 min. The supernatant was removed and the protein concentration was measured with BCA method. About 40 μg of protein was loaded each lane, and separated by 10% SDS-PAGE and then transferred to the PVDF membrane. The membrane was blocked by 5% non-fat milk powder for 1 h at room temperature before overnight incubation with primary antibodies 4°C, followed by the secondary antibody. The antibodies were rabbit anti-LIN28A (Cell Signaling, CAT 8641), rabbit anti-MeCP2 (Cell Signaling, CAT 3456), mouse anti-LIN28B (Cell Signaling, CAT 5422), mouse anti-β-Tubulin (Cell Signaling, CAT 6181), rabbit anti-OCT4 (Cell Signaling, CAT 2840), rabbit anti-SOX2 (Cell Signaling, CAT 3579), rabbit anti-NANOG (Cell Signaling, CAT 4903), rabbit anti-GAPDH (Cell Signaling, CAT 3683), rabbit anti-MBD3 (Cell Signaling, CAT 3896), rabbit anti-c-Myc (Cell Signaling, CAT 5605), mouse anti-β-actin (Cell Signaling, CAT 12262), mouse anti-MBD2 (Abcam, CAT ab45027), mouse anti-Flag (SIGMA, CAT F1804), rabbit anti-MMP2 (ImmunoWay, CAT YT2798), rabbit anti-MMP9 (ImmunoWay, CAT YT1892).

### Methylation analysis of LIN28A CpG islands

According to the MethyPrimer software, two potential CpG islands were predicted. The first CpG islands were identified in the *LIN28A* promoter region and the first exon from −79 bp to +98 bp, and the second CpG islands were in the first intron from +139 bp to +406 bp. We determined the methylation status of *LIN28A* CpG islands by bisulfite-sequencing. The genomic DNA was extracted from SW1990, PaTu8988, and PANC1 cells using TIANamp Genomic DNA kit (TIANGEN, CAT DP304) according to the manufacturer's recommendation, and 500 ng genomic DNA was treated with sodium bisulfite using EpiTect Bisulfite kit (QIAGEN, CAT 59104). 80-100 ng bisulfite-treated DNA was used for PCR amplification. The methylation status of the *LIN28A* CpG islands was determined by BSP using the specific primers designed according to the online primer program MethPrimer (http://www.urogene.org/methprimer/). Primer sequences were provided in Table 1. The length of the amplified sequence was 240 bp containing 13 CG sites, 267 bp, containing 18 CG sites, respectively. The total volume for BS-PCR was 10μl, with 5μl methylated DNA. The reaction procedure was as follows: 95°C 5 min, (95°C 30s, 53°C 30s, 72°C 30s) × 40 cycles, 72°C 10 min. The BSP products were cloned into pMD19-T vector (TaKaRa, CAT D102A) and then transformed in DH5α cells for clonal analyses. Each individual clone was sequenced by Sangon Company.

### 5-Aza-CdR treatment

The cells (SW1990, PaTu8988, and PANC1) were treated wi 5-Aza-CdR (Sigma, CAT A3656) in different doses (0, 1 and 2μmol/L) for 72h.

### Chromatin Immunoprecipitation (ChIP) assay

ChIP was performed using SimpleChIP Plus Enzymatic chromatin IP Kit (Agarose Beads) (Cell Signaling, CAT9004) according to the manufacturer's recommendations. Cells were fixed with formaldehyde and lysed, and chromatin was fragmented by partial digestion with Micrococcal Nuclease tomatic fragmentation to obtain chromatin fragments of 1 to 5 nucleosomes. Chromatin immunoprecipitations were performed using the MBDs, 10 μl of MeCP2, MBD3 antibodies and ChIP-Grade Protein G Agarose Beads. After reversal of protein-DNA cross-links, the DNA was purified using DNA purification spin columns.

### ChIP-qPCR

The enrichment of particular DNA sequences of SW1990, PaTu8988, and PANC1 cells were analyzed by quantitative PCR. The reaction mixture contains 2×SYBR-Green reaction mix 10μl, 5μM primers 2μl, nuclease-free water 6μl and the appropriate DNA sample 2μl. The 2% input chromatin DNA was diluted 5 times. Reactions were carried out in a thermal cycler under the following conditions: 95°C for 3 min followed by 95°C for 15 s, 60°C for 60 s for 40 cycles.

### Plasmid construction

The entire LIN28A sequence was amplified with RT-PCR using primers LIN28A-all-F and LIN28A-all-R (Table 1), and then cloned into the expression vector p3xFLAG-Myc-CMV™-24 (Sigma). The MeCP2, MBD3 and EGFP shRNA oligos (Table 1, Sangon Biotech., Shanghai) were firstly annealed into double strands and then cloned into pLKO.1-puro-vector (Sigma).

### Transfection

Cells were transfected with plasmid DNA or shRNAs using lipofectamine^TM^2000 Reagent (Invitrogen) following the manufacturer's protocol.

### Colony-forming assay

Transfected PANC1, PaTu8988 and SW1990 cells were harvested, resuspended in medium and transferred to the six well plate (500, 1000, 1500 cells per well) for 10-14 days until large colonies were visible. Colonies were fixed fixed and stained with 0.05% crystal violet for 30min, and the number of colonies was counted or photomicrographs were taken under phase-contrast microscope.

### Sphere formation assay

The sphere formation assay was performed by plating dissociated single cells at a density of 1 cell/μl in 6-well plates, and counting the number of spheres that formed after 7-14 days culture in stem cell medium [29].

#### Invasion assay

Invasion assays were carried out using matrigel chambers (BD Biosciences) according to the manufacturer's protocol. In brief, transfected PANC1, PaTu8988, BxPc3 and SW1990 cells were harvested, resuspended in serum-free medium and transferred to the hydrated matrigel chambers (∼10000 cells per well). The chambers were then incubated for 24h in culture medium with 10% FBS in the bottom chambers before examination. The cells on the upper surface were scraped and washed away, whereas the invaded cells on the lower surface were fixed and stained with 0.05% crystal violet for 30min. Finally, invaded cells were counted and the relative number was calculated.

### Data analysis

All the experiments were performed three times with triplicate samples. Comparisons between groups were analyzed using the Student's t test (two groups) or a one-way ANOVA (multiple groups). Differences with *P* values less than 0.05 were considered significant. DNA methylation data from bisulfite sequencing were analyzed and visualized using BiQ Analyzer.

## SUPPLEMENTARY MATERIAL FIGURES


