# Supplementary material for: MeCP2 suppresses LIN28A expression via binding to its methylated-CpG islands in pancreatic cancer cells
Source: Oncotarget. 2016 Feb 19;7(12):14476–85. doi: 10.18632/oncotarget.7507 (PMC4924729; doi:10.18632/oncotarget.7507)
Supplement: Supplementary file 1 [file oncotarget-07-14476-s001.pdf]

MeCP2 suppresses LIN28A expression *via* binding to its methylated-CpG islands in pancreatic cancer cells

Supplementary Material

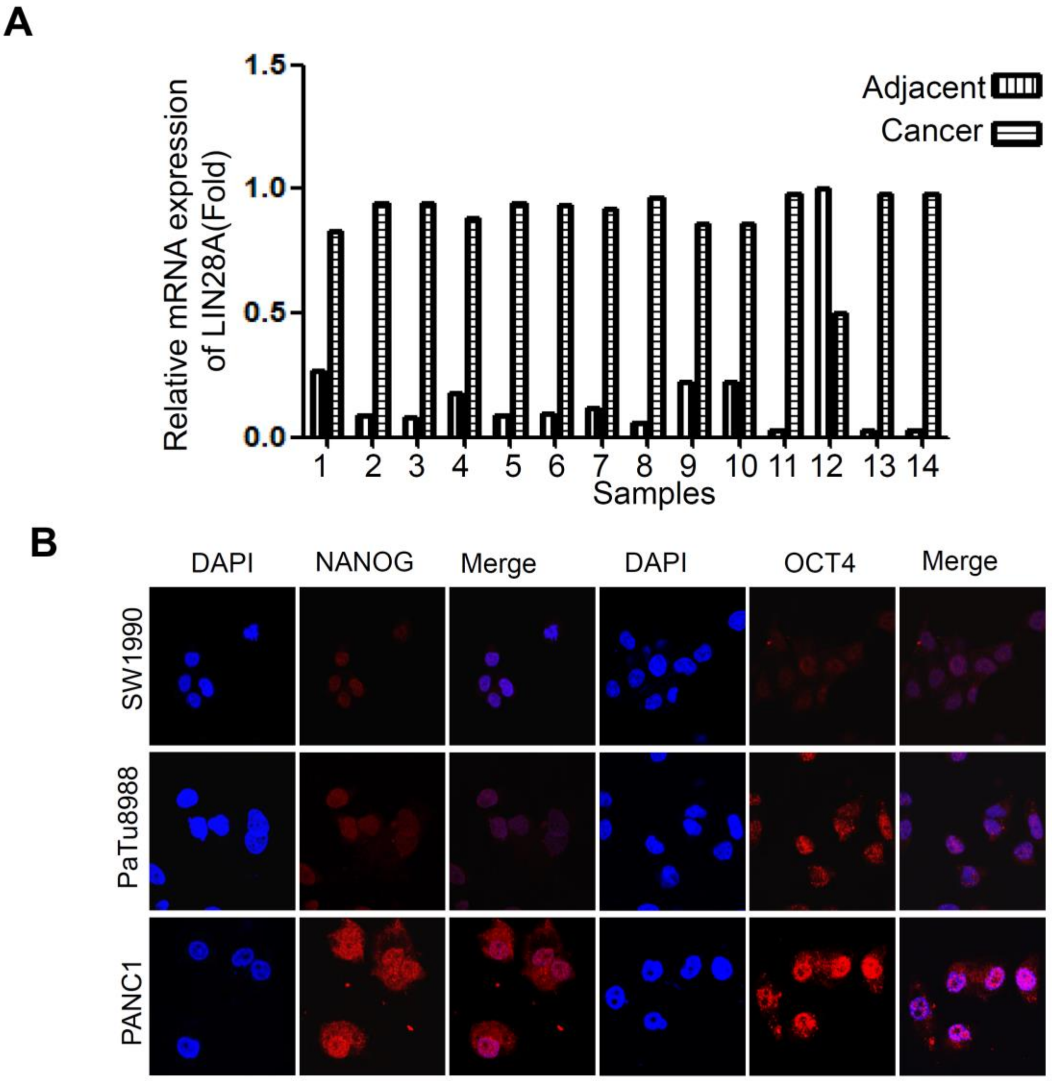

Fig.S1 (A) The LIN28A mRNA expression in pancreatic adjacent and cancer tissues. **(B)**The stem cell makers OCT4 (red), NANOG (red) and nuclei (DAPI, blue) were determined using immunofluorescence staining in SW1990, PaTu8988, and PANC1 cells. Scare bar = 50μm.

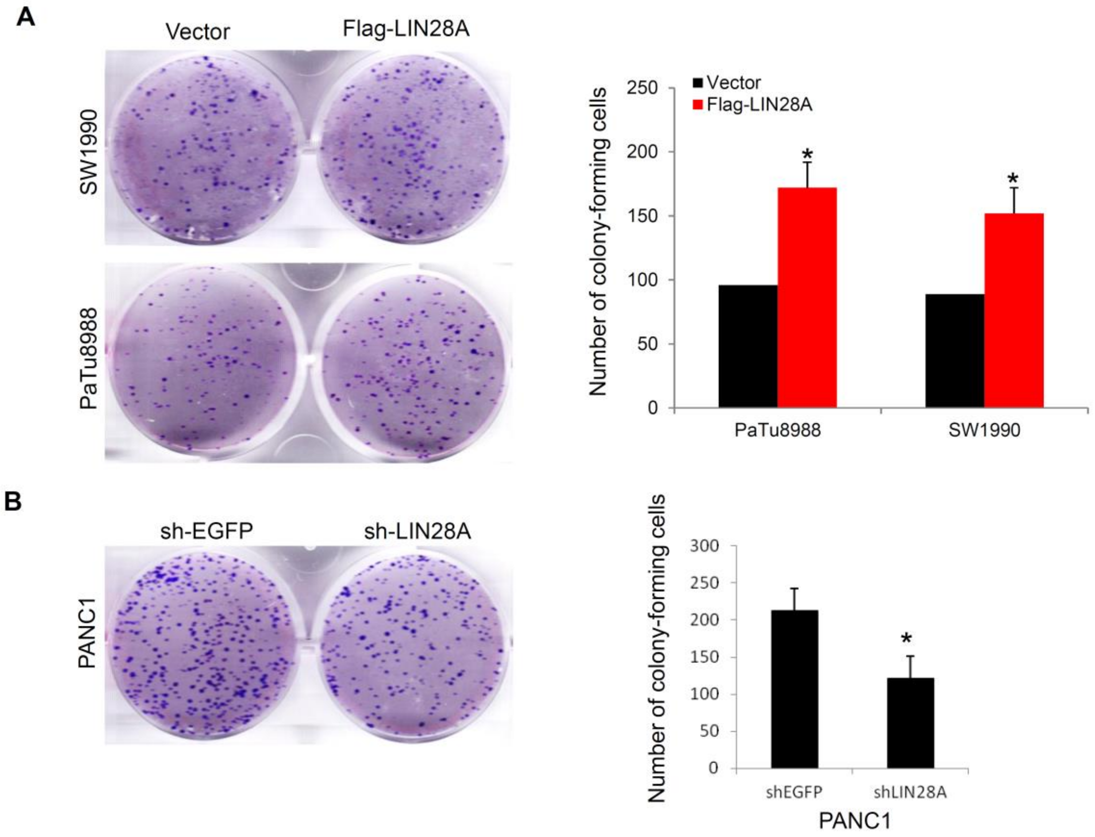

Fig.S2 LIN28A promotes the colony-forming of pancreatic cancer cells. (**A**) The abilities of colony-forming were examined using six-plate well in PaTu8988 and SW1990 cells transfected with Flag-LIN28A or Vector plasmids. The colony number was counted and analyzed in right. (**B**) PANC1 cells were transfected with sh-LIN28A or sh-EGFP plasmids, and Colonies were fixed and stained. Photomicrographs were taken under phase-contrast microscope (left), and the number of colonies was counted (right). Data shown are mean±SD of three triplicate measures. *, $P<0.05$.

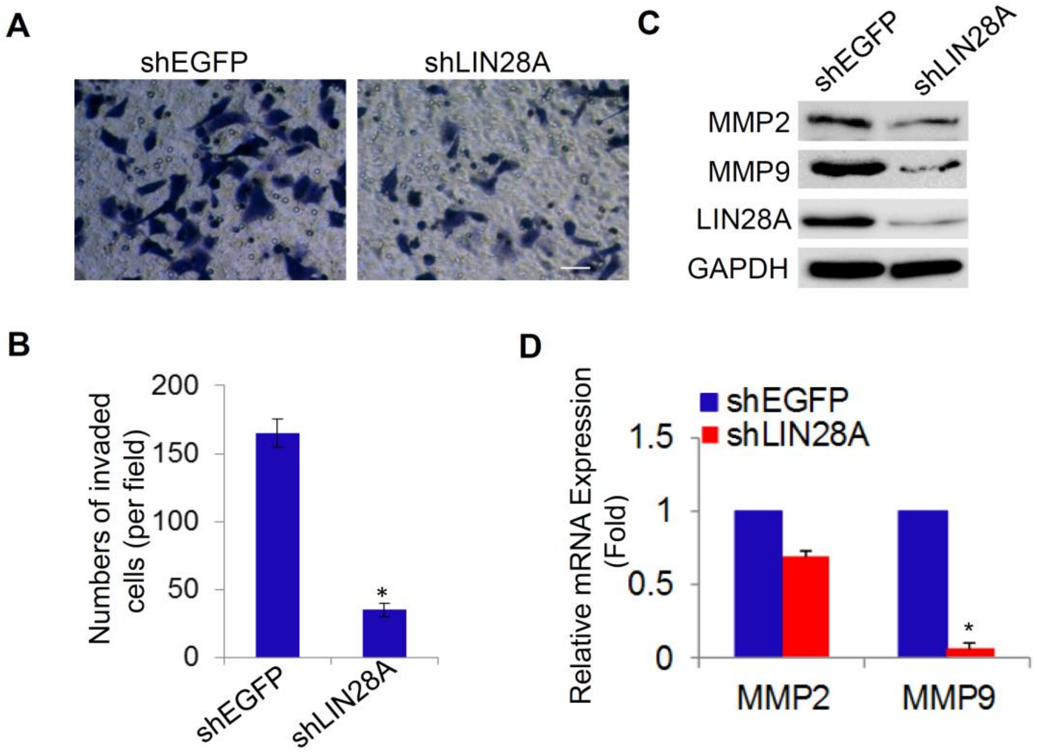

Fig.S3 LIN28A knockdown inhibits the invasiveness in PANC1 cells. (**A**) The abilities of invasiveness were examined using transwell assay in PANC1 cells transfected with sh-LIN28A or sh-EGFP plasmids. Invaded cells were counted and analyzed in (**B**). MMP2 and MMP9 were determined using western blotting **(C)** and real-time PCR **(D)** in above cells. Data shown are mean±SD of three triplicate measures. *, *P*<0.05.
